# Individualized Prediction of Survival Benefit From Locoregional Surgical Treatment for Patients With Metastatic Breast Cancer

**DOI:** 10.3389/fonc.2020.00148

**Published:** 2020-02-18

**Authors:** Yajuan Zheng, Guansheng Zhong, Kun Yu, Kefeng Lei, Qiong Yang

**Affiliations:** ^1^Department of Breast and Thyroid Surgery, Zhejiang Provincial People's Hospital, People's Hospital of Hangzhou Medical College, Hangzhou, China; ^2^Department of Breast Surgery, The First Affiliated Hospital, College of Medicine, Zhejiang University, Hangzhou, China; ^3^Department of General Surgery, The 7th Affiliated Hospital of Sun Yat-sen University, Shenzhen, China

**Keywords:** metastatic breast cancer, nomogram, SEER program, prognosis, clinic utility

## Abstract

**Objective:** Recently, performing locoregional surgical treatment still remains debatable in patients with metastatic breast cancer (MBC). Current study aimed to develop prognostic nomograms for predicting the long-term survival in MBC patients with or without surgical intervention, thereby assisting clinicians in making individualized choice.

**Methods:** The training set included 5173 patients who were diagnosed with MBC in 2010–2013 from the Surveillance, Epidemiology, and End Results Program, while the validation set comprised 2924 patients diagnosed in 2014–2015. Multivariant Cox hazard model was applied to determine the independent risk factors for overall survival (OS) and breast cancer specific survival (BCSS). Then, individualized pre- and postoperative nomograms for predicting 1- or 3-year survival probabilities were constructed accordingly. Internal and external validations were conducted to determine the accuracy of these nomograms by calculating concordance index (C-index) and plotting calibration curves.

**Results:** The survival analysis indicated that surgical management conferred improved OS and BCSS in patients with metastatic breast cancer. Age, T stage, grade, distant metastatic site, ER, PR and HER2 status, radiation, and chemotherapy were independent risk factors for OS and BCSS both in surgery and non-surgery group. All these factors were subsequently incorporated into the nomogram which showed acceptable predictive capabilities with C-index range of 0.65–0.80 both in training set and external validation set. In addition, a preoperative nomogram incorporating variables capable of being determined before surgery was also built with C-index above 0.70 both in training and validation set.

**Conclusion:** Surgical management in patients with metastatic breast cancer suggests a potential survival advantage. In addition, these well-validated pre- and postoperative nomograms may provide a useful tool to assist clinicians in treatment decision-making and in evaluating patients' long term prognosis.

## Introduction

Breast cancer is the most frequently diagnosed cancer in women, and accounts for the second leading cause of cancer-related mortality in the USA (1). Although the treatment of breast cancer has made great progress in recent years, largely because of the emergence of endocrine therapy and anti-HER2 therapy, surgical treatment is still the preferred option for non-metastatic breast cancer and is considered the foundation of subsequent comprehensive treatment. Nevertheless, a substantial proportion of breast cancer patients, approximately 6%, have suffered distant metastasis when they are first diagnosed (2). It was reported that the median survival time of metastatic breast cancer (MBC) patients is approximately 18–24 months with 5– and 10–year survival rates as low as 27 and 13%, respectively (3).

Since stage IV breast cancer is still considered incurable, the primary goal of treatment is to extend life expectancy and improve quality of life. According to the NCCN guideline, the primary treatment approach for metastatic breast cancer is systemic therapy, and surgery is not recommended except for those patients requiring palliation of symptoms or with impending complications, such as skin ulceration and bleeding (4). However, although MBC might exhibit good response to systemic therapy, like chemotherapy and endocrine therapy, the majority of patients suffered disease progression after 1–2 years (5). Over the past several years, some retrospective studies have suggested a potential survival benefit from aggressive surgical excision of primary breast tumor in patients with metastatic breast cancer (6–9). However, several studies have also indicated that surgical intervention does not improve survival of patients with metastatic breast cancer (10, 11). A prospective clinical trials conducted in India (NCT00193778) demonstrated that locoregional treatment of the primary tumor does not affect overall survival in MBC patients (12). On the contrary, another prospective study named MF07-01 (NCT00557986) in Turkey reported that the initial surgery group showed statistically significant improvement in 5-year overall survival, especially in subgroup with positive hormone receptors (HR), negative HER2, or younger than age 55 (13). They hold the opinion that various factors including age, comorbidities, tumor type and metastatic disease burden should be considered before opting locoregional treatment in *de novo* stage IV breast cancer. Moreover, after combination of those two randomized clinical trials, a recent systemic review concluded that existing evidence was insufficient to make definitive conclusions on the survival benefit of breast surgery for patients diagnosed with MBC (14). Recently, clinicians still remain ambivalent about whether to perform primary tumor surgery for patients with MBC. Therefore, a more individualized approach considering potential risks and benefits of surgical intervention may be justified.

As such, this study exploited the data from SEER program to separately identify independent prognostic factors associated with survival of MBC patients who received surgical treatment or not. Several individualized nomograms were subsequently constructed for predicting the long term survival of MBC patients with or without surgery. We also designed a preoperative version of nomogram in which each factor can be determined before surgery decision. After that, those nomograms were separately validated in an external dataset. We hope that those nomograms may assist clinicians in evaluating each patient's long term survival by taking multiple risk factors into consideration, thereby allowing for more personalized stratification of the potential benefits of surgical intervention for patients suffered from metastatic breast cancer.

## Materials and Methods

### Database and Patient Selection

**Figure 1:**
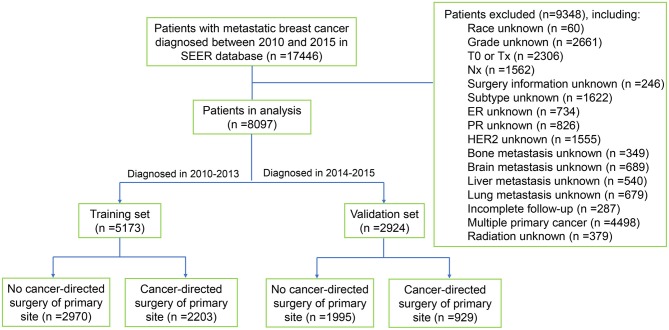
Flowchart of data selection. SEER, Surveillance, Epidemiology, and End Results Program.

Data were extracted from the recently released SEER database [Incidence- SEER 18 Regs Custom Data (with additional treatment fields), Nov 2018 Sub] containing information of cancer patients diagnosed from 1975 to 2016. SEER^*^Stat software version 8.3.6 (National Cancer Institute, USA) was used to access the database with permission from the SEER program office. A total of 17446 patients met the criteria of metastatic breast cancer (International Classification of Diseases for Oncology- 3 histologic type/behavior code: 8500/3-8543/3) who were diagnosed from 2010 to 2015 were screened out from the database. Subsequently, patients who met the following criteria were excluded: (1) unknown race; (2) unknown histological grade; (3) stage T0, TX or NX breast cancer; (4) unknown specific surgery type; (5) unknown estrogen receptor (ER), progesterone receptor (PR), or HER2 status; (6) unknown information of distant metastasis; (7) unknown radiation information; (8) patients with incomplete follow-up; (9) patients with multiple primary cancer. Finally, 8097 metastatic breast cancer patients were included in this study. Of these patients, 5173 patients who were diagnosed from 2010 to 2013 were chosen as the training set, while 2924 patients diagnosed from 2014 to 2015 were used as the validation cohort. Subsequently, each cohort was further divided into two subgroups based on whether they had undergone locoregional surgical treatment or not. The flowchart of patient selection was shown in Figure 1.

### Covariates

Variables including demographic characteristics (age at diagnosis, gender, Race), disease characteristics (T stage, N stage, histological grade, distant metastatic site, ER, PR and HER2 status), and treatment characteristics (radiation, chemotherapy, and surgery type) were involved in the analysis. Continuous variable, age at diagnosis, was transformed into categorical variables (<35, 35–49, 50–69, and ≥70). Based on specific surgery information, surgery type was categorized into two groups, lumpectomy/mastectomy (lumpectomy, subcutaneous mastectomy, or total mastectomy) and radical mastectomy (radical mastectomy, modified radical mastectomy, or extended radical mastectomy). Survival months, vital status record, and cause-specific death classification were used to calculate OS and BCSS.

### Statistical Analysis

Descriptive statistics were first used to assess the baseline characteristics of metastatic breast cancer patients. Chi-square test was utilized to compare the clinicopathologic characteristics between the training and validation set. Kaplan-Meier plot and log-rank test were performed to compare differences of OS and BCSS between surgery and non-surgery group. For subgroup analyses, a multivariate Cox hazard model containing all covariates, including age, T and N stage, histological grade, distant metastatic site, ER, PR and HER2 status, record of radiation and chemotherapy, was utilized to evaluate the survival benefit of locoregional surgical treatment in each subgroup. For subsequent survival analysis in subgroups with or without surgery, univariate Cox proportional hazard model was first generated to estimate the impact of each variable on OS and BCSS. Then, all variables with p-value < 0.05 in univariate Cox model were included in multivariate Cox proportional hazard model.

Individualized nomograms for both surgery and non-surgery subgroups were developed to predict 1- or 3-year OS and BCSS according to the multivariate Cox result. Since predicting survival preoperatively makes great sense with regard to the surgical decision-making, a new version of preoperative nomogram was also constructed by including covariates that can be evaluated preoperatively either by needle biopsy or advanced imaging method, including age, T and N stage, ER, PR and HER2 status, histological grade, and distant metastatic site. The accuracies of these nomograms were evaluated by means of discrimination and calibration. Discrimination was measured using the concordance index (C-index), while calibration was assessed by graphic calibration curves which estimate the consistency between the nomogram predicted probability and actual observed outcome. We also evaluated these nomograms in the external validation set by calculating the C-index and plotting the calibration curves. All the statistical analyses were performed using SPSS 24.0 (Chicago, IL, USA). All the nomograms and calibration curves were plotted by using R software version 3.6.0. A two-tailed *p* < 0.05 was considered statistically significant.

## Results

### Characteristics of Patients in the Datasets

Through rigorous screening and selection, a total of 8097 patients with metastatic breast cancer diagnosed from 2010 to 2015 were included in this study. All these patients were divided into training and validation set for the purpose of performing an external validation. The training set included 5173 patients diagnosed from 2010 to 2013, while the validation set comprised 2924 patients diagnosed form 2014 to 2015. The baseline characteristics of these two cohorts were shown in Supplementary Table 1. The proportion of breast cancer patients who had undergone surgery treatment in validation set was relatively lower (31.8 vs. 42.6 %) than training set. Moreover, patients in validation set had received less radiation therapy than training set (61.9 vs. 66.8 %). In general, the characteristics of the patients in validation set were slightly different compared with the training set, implying a higher value of external validation.

Among the 5173 breast cancer patients in training set, 2203 patients had received locoregional surgical treatment while 2970 patients had not undergone cancer directed surgery. As shown in Table 1, patients in the surgery group had higher proportion of 35–49-year-old age (24.6 vs. 17.8 %) compared with non-surgery group (*p* < 0.001). Patients in surgery group tended to have tumor with smaller size, higher histological grade, hormone-receptor (HR) positive, and more extent of regional lymph node involvement (all *p* < 0.05). Moreover, the non-surgery group was more likely to suffer multiple distant metastasis (40.3 vs. 19.4%), and was less likely to receive radiation (30.1 vs. 49.0%) and chemotherapy (54.0 vs. 69.8%) than surgery group (all *p* < 0.001).

**Table 1:** Patient demographics and disease characteristics.

**Variables**	**No surgery (*n* = 2970), n (%)**	**Surgery (*n* = 2203) n (%)**	***p*-value**
Age[median (IQR^a^)]	60 (51–70)	57 (48–67)	*p < * 0.001
<35	99 (3.3)	102 (4.6)	*p < * 0.001
35–49	528 (17.8)	542 (24.6)	
50–69	1562 (52.6)	1124 (51.0)	
≥ 70	781 (26.3)	435 (19.7)	
**Gender**			
Female	2941 (99.0)	2172 (98.6)	*p =* 0.153
Male	29 (1.0)	31 (1.4)	
**Race**			
Black	552 (17.6)	363 (16.5)	*p =* 0.542
White	2216 (74.6)	1672 (75.9)	
Other	232 (7.8)	168 (7.6)	
**T Stage**			
T1	351 (11.8)	261 (11.8)	*p < * 0.001.
T2	928 (31.2)	857 (38.9)	
T3	504 (17.0)	421 (19.1)	
T4	1187 (40.0)	664 (30.1)	
**N Stage**			
N0	721 (24.3)	369 (16.7)	*p < * 0.001
N1	1569 (52.8)	829 (37.6)	
N2	287 (9.7)	449 (20.4)	
N3	393 (13.2)	556 (25.2)	
**Grade**			
High, I	225 (7.6)	131 (5.9)	*p < * 0.001
Intermediate, II	1335 (44.9)	744 (33.8)	
Low, III	1385 (46.6)	1311 (59.5)	
Anaplastic, IV	25 (0.8)	17 (0.8)	
**Distant Metastasis**			
Bone only	1024 (34.5)	920 (41.8)	*p < * 0.001
Liver only	191 (6.4)	206 (9.4)	
Lung only	264 (8.9)	251 (11.4)	
Brain only	32 (1.1)	22 (1.0)	
Other site	261 (8.8)	376 (17.1)	
Multiple sites	1198 (40.3)	428 (19.4)	
**ER Status**			
Negative	723 (24.3)	636 (28.9)	*p < * 0.001
Positive	2247 (75.7)	1567 (71.1)	
**PR Status**			
Negative	1164 (39.2)	940 (42.7)	*p =* 0.012
Positive	1806 (60.8)	1263 (57.3)	
**HER2 Status**			
Negative	2167 (73.0)	1597 (72.5)	*p =* 0.707
Positive	803 (27.0)	606 (27.5)	
**Axillary Lymph Node**			
Negative	41 (1.4)	299 (13.6)	*p < * 0.001
Positive	832 (28.0)	1529 (69.4)	
Not evaluated	2097 (70.6)	375 (17.0)	
**Radiation**			
No	2077 (69.9)	1123 (51.0)	*p < * 0.001
Yes	893 (30.1)	1080 (49.0)	
**Chemotherapy**			
No	1367 (46.0)	666 (30.2)	*p < * 0.001
Yes	1603 (54.0)	1537 (69.8)	

a *interquartile range*.

### Analysis of Survival Benefits From Surgery

**Figure 2:**
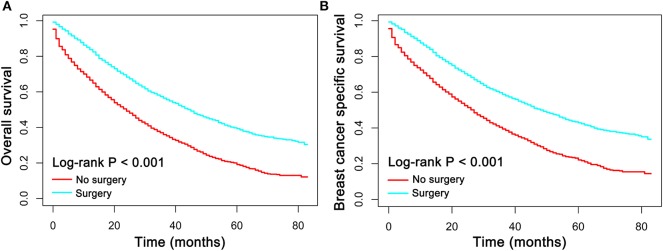
**(A)** Overall survival (OS) and **(B)** breast cancer specific survival (BCSS) curves plotted by Kaplan-Meier method for patients received surgical treatment or not.

It has been recommended by the NCCN guideline that the primary treatment approach for women with metastatic breast cancer is systemic therapy rather than surgical treatment. In order to evaluate the survival benefits of local breast surgery in patients with metastatic breast cancer, the Kaplan-Meier plot was performed to compare the OS and BCSS between patients who had, or had not undergone local breast surgical treatment. The median follow-up duration in the training set was 30 months (mean, 31.1 month; range, 0 to 83 months). Of all the 5173 patients with metastatic breast cancer, a total of 1947 patients were dead at the time of last follow-up and 1643 of which were dead directly from breast cancer. As shown in Figure 2, patients who had undergone surgical treatment had prominently better OS and BCSS than patients who had not (*p* < 0.001).

In order to determine if metastatic breast cancer patients in specific subgroup could benefit from surgical treatment, subgroup analysis stratified based on age, disease characteristics, and treatment were conducted. As shown in Table 2, the results of multivariant Cox analysis demonstrated that local surgical treatment exerted a significant survival benefit both in OS and BCSS in almost all subgroups (*p* < 0.05) except in the patients with undifferentiated breast cancer (*p* > 0.05). These results, taken together, indicated that locoregional surgical treatment was significantly associated with improved OS and BCSS in patients with metastatic breast cancer.

**Table 2:** Subgroup analysis of OS and BCSS outcomes.

**Variables**	**OS**		**BCSS**	
	**HR (95% CI)**	***p*-value^a^**	**HR (95% CI)**	***p*-value**
**Age**				
<35	0.38 (0.24–0.61)	<0.001	0.38 (0.23–0.62)	<0.001
35–49	0.61 (0.51–0.73)	<0.001	0.62 (0.52–0.75)	<0.001
50–69	0.54 (0.48–0.61)	<0.001	0.55 (0.49–0.62)	<0.001
≥ 70	0.62 (0.53–0.72)	<0.001	0.66 (0.57–0.76)	<0.001
**T Stage**				
T1	0.46 (0.36–0.60)	<0.001	0.45 (0.35–0.59)	<0.001
T2	0.53 (0.47–0.61)	<0.001	0.55 (0.48–0.63)	<0.001
T3	0.57 (0.48–0.68)	<0.001	0.57 (0.47–0.69)	<0.001
T4	0.66 (0.59–0.75)	<0.001	0.64 (0.56–0.73)	<0.001
***N*** **Stage**				
N0	0.52 (0.44–0.61)	<0.001	0.53 (0.44–0.63)	<0.001
N1	0.55 (0.49–0.62)	<0.001	0.55 (0.48–0.62)	<0.001
N2	0.54 (0.44–0.65)	<0.001	0.53 (0.43–0.65)	<0.001
N3	0.72 (0.60–0.85)	<0.001	0.70 (0.58–0.83)	<0.001
**Grade**				
High, I	0.55 (0.39–0.77)	<0.001	0.45 (0.31–0.65)	<0.001
Intermediate, II	0.56 (0.49–0.64)	<0.001	0.56 (0.48–0.64)	<0.001
Low, III	0.60 (0.54–0.66)	<0.001	0.60 (0.54–0.67)	<0.001
Undifferentiated, IV	0.55 (0.16–1.86)	0.334	0.86 (0.23–3.19)	0.820
**Distant Metastasis**				
Bone only	0.51 (0.45–0.58)	<0.001	0.52 (0.45–0.59)	<0.001
Liver only	0.75 (0.57–0.99)	0.041	0.74 (0.56–0.99)	0.042
Lung only	0.58 (0.46–0.73)	<0.001	0.53 (0.41–0.67)	<0.001
Brain only	0.31 (0.14–0.69)	0.004	0.29 (0.13–0.65)	0.003
Other site	0.59 (0.46–0.75)	<0.001	0.55 (0.42–0.71)	<0.001
Multiple sites	0.61 (0.53–0.70)	<0.001	0.62 (0.54–0.71)	<0.001
**ER Status**				
Negative	0.54 (0.47–0.62)	<0.001	0.56 (0.49–0.64)	<0.001
Positive	0.60 (0.55–0.66)	<0.001	0.58 (0.53–0.64)	<0.001
**PR Status**				
Negative	0.63 (0.56–0.70)	<0.001	0.64 (0.57–0.71)	<0.001
Positive	0.55 (0.49–0.61)	<0.001	0.53 (0.47–0.59)	<0.001
**HER2 Status**				
Negative	0.59 (0.54–0.64)	<0.001	0.58 (0.53–0.64)	<0.001
Positive	0.52 (0.44–0.61)	<0.001	0.54 (0.45–0.64)	<0.001
**Radiation**				
No	0.68 (0.62–0.75)	<0.001	0.68 (0.62–0.75)	<0.001
Yes	0.43 (0.38–0.49)	<0.001	0.43 (0.38–0.49)	<0.001
**Chemotherapy**				
No	0.61 (0.54–0.69)	<0.001	0.60 (0.53–0.68)	<0.001
Yes	0.56 (0.50–0.62)	<0.001	0.56 (0.51–0.63)	<0.001

a *Multivariant Cox regression model*.

*All HRs refer to Surgery versus non-surgery in the subgroup analysis*.

*CI, confidence interval; BCSS, breast cancer specific survival*.

### Risk Covariates Related With Survival in Cohorts With and Without Surgery

Initially, univariate Cox proportional models regarding to groups with and without surgery were built, respectively, to evaluate the multiple factors related with OS and BCSS (Table 3). Eleven parameters were incorporated into this Cox model, including one demographic variable, seven disease-related variables, and three treatment-related variables. As shown in Table 3, the risk of death increased dramatically with age both in cohort with and without surgery. The T staging exerted a significant prognostic factors. For patients not receiving surgery, the risk of death in patients with higher T stage (≥T3) was higher than those with T1 tumors expect T2 tumors. Meanwhile, among patients receiving surgery, T staging (≥T2) was consistently associated with worse OS (T2 vs. T1, HR = 1.33, 95% CI [1.08-1.64]; T3 vs. T1, HR = 1.54, 95% CI [1.23-1.93]; T4 vs. T1, HR = 2.13, 95% CI [1.73-2.62]) and BCSS (T2 vs. T1, HR = 1.37, 95% CI [1.10-1.70]; T3 vs. T1, HR = 1.53, 95% CI [1.21-1.94]; T4 vs. T1, HR = 2.11, 95% CI [1.70-2.63]) compared with T1 (all p < 0.05). The risk of death also increased in patients with poorer tumor differentiation. Patients with lung, brain or multiple sites involvement had a significantly higher risk of death than those with only bone metastases regardless of surgery or not (all *p* < 0.05). However, there was no correlation between higher N staging and poorer survival outcomes in both groups. Moreover, positive status of ER, PR and HER2, and treatments with radiation and chemotherapy were proved to be protective factors for better OS and BCSS in both surgery and non-surgery group. Intriguingly, patients received radical mastectomy had slightly better prognosis than those undergone lumpectomy or mastectomy both in OS (HR = 1.16, 95% CI [1.04-1.29], p = 0.009) and BCSS (HR = 1.15, 95% CI [1.02-1.29], p = 0.019).

**Table 3:** Univariant Cox models for metastasis breast cancer patients in surgery and non-surgery set.

**Variables**	**OS**				**BCSS**			
	**No surgery**		**Surgery**		**No surgery**		**Surgery**	
	**HR (95% CI)**	***p*-value**	**HR (95% CI)**	***p*-value**	**HR (95% CI)**	***p*-value**	**HR (95% CI)**	***p*-value**
Age		<0.001		<0.001		<0.001		<0.001
<35	Reference		Reference		Reference		Reference	
35–49	1.06 (0.81–1.38)	0.668	1.38 (0.99–1.93)	0.061	1.08 (0.82–1.42)	0.587	1.48 (1.04–2.11)	0.028
50–69	1.32 (1.03–1.70)	0.029	1.71 (1.24–2.37)	0.001	1.33 (1.02–1.73)	0.034	1.78 (1.27–2.50)	0.001
≥70	1.81 (1.40–2.34)	<0.001	2.74 (1.96–3.82)	<0.001	1.72 (1.31–2.25)	<0.001	2.55 (1.79–3.62)	<0.001
T stage		<0.001		<0.001		<0.001		<0.001
T1	Reference	<0.001	Reference		Reference	<0.001	Reference	
T2	1.02 (0.88–1.17)	0.842	1.33 (1.08–1.64)	0.008	1.03 (0.88–1.20)	0.732	1.37 (1.10–1.70)	0.005
T3	1.18 (1.01–1.39)	0.037	1.54 (1.23–1.93)	<0.001	1.21 (1.03–1.43)	0.024	1.53 (1.21–1.94)	<0.001
T4	1.32 (1.15–1.51)	<0.001	2.13 (1.73–2.62)	<0.001	1.35 (1.17–1.56)	<0.001	2.11 (1.70–2.63)	<0.001
N stage		0.095		0.003		0.226		0.027
N0	Reference		Reference		Reference		Reference	
N1	0.91 (0.83–1.01)	0.081	0.86 (0.73–1.01)	0.074	0.95 (0.85–1.05)	0.317	0.88(0.74–1.05)	0.148
N2	1.07 (0.91–1.24)	0.415	1.02 (0.85–1.22)	0.866	1.09 (0.93–1.28)	0.286	1.01 (0.84–1.22)	0.888
N3	0.97 (0.85–1.12)	0.715	1.12 (0.95–1.33)	0.188	1.02 (0.88–1.18)	0.794	1.10 (0.92–1.32)	0.291
Grade		<0.001		<0.001		<0.001		<0.001
High, I	Reference		Reference		Reference		Reference	
Intermediate, II	1.26 (1.06–1.50)	0.008	1.26 (0.95–1.68)	0.108	1.28 (1.07–1.54)	0.008	1.45 (1.06–2.00)	0.021
Low, III	1.73 (1.46–2.05)	<0.001	2.08 (1.58–2.73)	<0.001	1.82 (1.52–2.18)	<0.001	2.46 (1.81–3.35)	<0.001
Anaplastic, IV	2.18 (1.40–3.42)	0.001	1.73 (0.88–3.40)	<0.111	2.14 (1.32–3.46)	0.002	2.21 (1.11–4.40)	0.024
Distant metastasis		<0.001		<0.001		<0.001		<0.001
Bone only	Reference		Reference		Reference		Reference	
Liver only	1.08 (0.90–1.30)	0.417	1.53 (1.25–1.86)	<0.001	1.11 (0.91–1.34)	0.309	1.51 (1.23–1.86)	<0.001
Lung only	1.26 (1.08–1.48)	0.004	1.49 (1.25–1.79)	<0.001	1.30 (1.10–1.53)	0.002	1.43 (1.18–1.73)	<0.001
Brain only	3.01 (2.07–4.37)	<0.001	3.05 (1.91–4.89)	<0.001	3.36 (2.31–4.88)	<0.001	2.94 (1.79–4.85)	<0.001
Other site	1.11 (0.94–1.30)	0.217	1.05 (0.89–1.25)	0.548	1.09 (0.92–1.29)	0.327	1.01 (0.84–1.20)	0.941
Multiple sites	1.62 (1.47–1.79)	<0.001	2.02 (1.75–2.32)	<0.001	1.68 (1.52–1.86)	<0.001	2.08 (1.80–2.41)	<0.001
**ER status**								
Negative	Reference		Reference		Reference		Reference	
Positive	0.54 (0.49–0.59)	<0.001	0.54 (0.48–0.61)	<0.001	0.53 (0.48–0.58)	<0.001	0.51 (0.45–0.58)	<0.001
**PR status**								
Negative	Reference		Reference		Reference		Reference	
Positive	0.60 (0.55–0.66)	<0.001	0.51 (0.46–0.57)	<0.001	0.58 (0.53–0.63)	<0.001	0.48 (0.42–0.53)	<0.001
**HER2 status**								
Negative	Reference		Reference		Reference		Reference	
Positive	0.79 (0.72–0.87)	<0.001	0.56 (0.49–0.65)	<0.001	0.80 (0.73–0.89)	<0.001	0.58 (0.51–0.67)	<0.001
**Radiation**								
No	Reference		Reference		Reference		Reference	
Yes	1.12 (1.02–1.22)	0.016	0.71 (0.64–0.79)	<0.001	1.14 (1.04–1.25)	0.006	0.73 (0.65–0.82)	<0.001
**Chemotherapy**								
No	Reference		Reference		Reference		Reference	
Yes	0.80 (0.74–0.87)	<0.001	0.76 (0.67–0.85)	<0.001	0.78 (0.72–0.85)	<0.001	0.80 (0.71–0.91)	<0.001
**Surgery type**								
Lumpectomy/mastectomy	–		Reference		–		Reference	
Radical mastectomy	–		1.16 (1.04–1.29)	0.009	–		1.15 (1.02–1.29)	0.019

In order to eliminate possible bias, all the aforementioned variables with *p* < 0.05 in univariate Cox analysis were enlisted into multivariate analysis. The detailed results of multivariate Cox analysis were shown in Table 4. Notably, nine variables (age, T stage, grade, distant metastatic site, ER, PR and HER2 status, radiation, and chemotherapy) remained significantly associated with survival outcome in both groups (*p* < 0.05). However, in surgery group, radical mastectomy no longer exerted as protective factor for improved OS as well as BCSS compared with lumpectomy/mastectomy.

**Table 4:** Multivariable Cox models for metastasis breast cancer patients in surgery and non–surgery set.

**Variables**	**OS**				**BCSS**			
	**No surgery**		**Surgery**		**No surgery**		**Surgery**	
	**HR (95% CI)**	***p*-value**	**HR (95% CI)**	***p*-value**	**HR (95% CI)**	***p*-value**	**HR (95% CI)**	***p*-value**
Age		<0.001		<0.001		<0.001		<0.001
<35	Reference		Reference		Reference		Reference	
35–49	1.02 (0.78–1.33)	0.888	1.24 (0.89–1.75)	0.221	1.03 (0.78–1.36)	0.817	1.35 (0.94–1.92)	0.100
50–69	1.24 (0.96–1.60)	0.097	1.36 (0.98–1.89)	0.059	1.24 (0.95–1.61)	0.112	1.43 (1.01–2.02)	0.042
≥ 70	1.58 (1.21–2.05)	0.001	2.05 (1.45–2.88)	<0.001	1.50 (1.14–1.97)	0.004	1.93 (1.35–2.77)	<0.001
T stage		<0.001		<0.001		<0.001		<0.001
T1	Reference		Reference		Reference	<0.001	Reference	
T2	1.01 (0.87–1.16)	0.946	1.22 (0.99–1.51)	0.121	1.01 (0.87–1.18)	0.903	1.24 (0.99–1.54)	0.059
T3	1.14 (0.97–1.33)	0.115	1.36 (1.08–1.72)	0.011	1.15 (0.97–1.36)	0.105	1.32 (1.04–1.69)	0.024
T4	1.22 (1.06–1.41)	0.005	1.80 (1.45–2.25)	<0.001	1.24 (1.07–1.44)	0.004	1.75 (1.39–2.20)	<0.001
N stage				0.037		–		0.160
N0	–		Reference		–	–	Reference	
N1	–		0.87 (0.74–1.04)	0.118	–	–	0.90 (0.75–1.07)	0.241
N2	–		0.99 (0.81–1.19)	0.872	–	–	0.98 (0.81–1.20)	0.871
N3	–		1.08 (0.90–1.29)	0.438	–	–	1.07 (0.88–1.29)	0.506
Grade		<0.001		<0.001		<0.001		<0.001
High, I	Reference		Reference		Reference		Reference	
Intermediate, II	1.28 (1.07–1.52)	0.006	1.25 (0.94–1.66)	0.121	1.29 (1.07–1.55)	0.008	1.44 (1.05–1.98)	0.026
Low, III	1.68 (1.40–2.01)	<0.001	1.81 (1.36–2.40)	<0.001	1.74 (1.44–2.10)	<0.001	2.10 (1.52–2.87)	<0.001
Anaplastic, IV	2.14 (1.36–3.37)	0.001	1.73 (0.88–3.43)	0.115	2.05 (1.26–3.35)	0.004	2.24 (1.11–4.49)	0.024
Distant metastasis		<0.001		<0.001		<0.001		<0.001
Bone only	Reference		Reference		Reference		Reference	
Liver only	1.11 (0.92–1.35)	0.287	1.66 (1.35–2.04)	<0.001	1.13 (0.92–1.38)	0.250	1.57 (1.26–1.94)	<0.001
Lung only	1.02 (0.87–1.20)	0.808	1.07 (0.88–1.29)	0.514	1.05 (0.88–1.24)	0.595	0.99 (0.81–1.21)	0.942
Brain only	2.39 (1.63–3.49)	<0.001	2.44 (1.51–3.92)	<0.001	2.58 (1.76–3.77)	<0.001	2.23 (1.34–3.69)	0.002
Other site	0.93 (0.79–1.10)	0.410	0.90 (0.76–1.08)	0.256	0.91 (0.77–1.09)	0.317	0.83 (0.69–1.00)	0.055
Multiple sites	1.57 (1.42–1.74)	<0.001	1.78 (1.53–2.06)	<0.001	1.62 (1.46–1.80)	<0.001	1.82 (1.56–2.12)	<0.001
**ER status**								
Negative	Reference		Reference		Reference		Reference	
Positive	0.57 (0.50–0.64)	<0.001	0.78 (0.66–0.91)	0.002	0.58 (0.51–0.67)	<0.001	0.76 (0.64–0.89)	0.001
**PR status**								
Negative	Reference		Reference		Reference		Reference	
Positive	0.71 (0.63–0.80)	<0.001	0.54 (0.46–0.63)	<0.001	0.68 (0.60–0.76)	<0.001	0.51 (0.43–0.59)	<0.001
**HER2 status**								
Negative	Reference		Reference		Reference		Reference	
Positive	0.61 (0.55–0.68)	<0.001	0.43 (0.37–0.49)	<0.001	0.60 (0.53–0.67)	<0.001	0.43 (0.37–0.50)	<0.001
**Radiation**								
No	Reference		Reference		Reference		Reference	
Yes	1.10 (1.01–1.21)	0.037	0.79 (0.71–0.89)	<0.001	1.12 (1.02–1.24)	0.020	0.80 (0.71–0.90)	<0.001
**Chemotherapy**								
No	Reference		Reference		Reference		Reference	
Yes	0.68 (0.62–0.75)	<0.001	0.74 (0.64–0.85)	<0.001	0.69 (0.62–0.76)	<0.001	0.74 (0.64–0.86)	<0.001
**Surgery type**								
Lumpectomy/mastectomy	–		Reference		–		Reference	
Radical mastectomy	–		1.10 (0.98–1.24)	0.117	–		1.10 (0.97–1.25)	0.136

### Individualized Construction of Nomogram and External Validation

**Figure 3:**
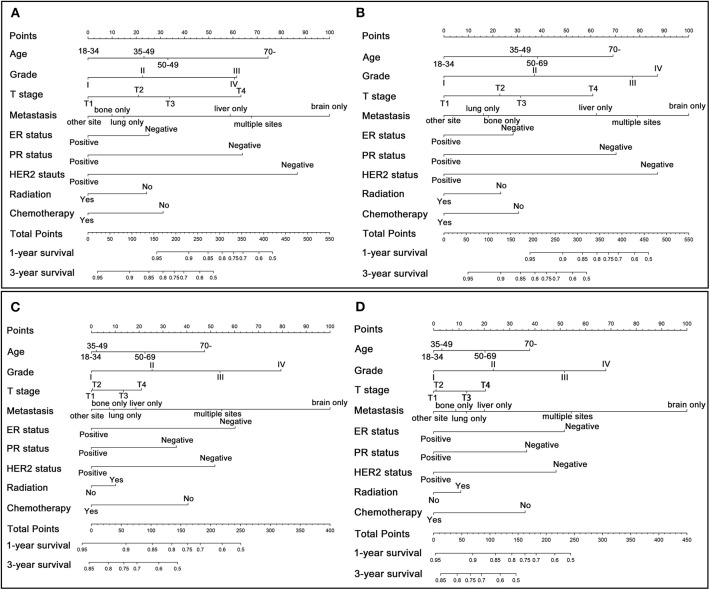
Nomogram for predicting 1- and 3-year OS and BCSS in patients with metastatic breast cancer. **(A)** OS for patients who undergo surgical treatment. **(B)** BCSS for patients who undergo surgical treatment. **(C)** OS for patients who does not undergo surgical treatment. **(D)** BCSS for patients who does not undergo surgical treatment.

According to the results of multivariate Cox analysis, separate nomograms were plotted to predict the 1- and 3-year OS and BCSS among patients with or without surgery (Figure 3). Since N staging and surgery type exerted no statistical significance in multivariate analysis (*p* > 0.05), nine variables (age, T stage, grade, distant metastatic site, ER, PR and HER2 status, radiation, and chemotherapy) were finally incorporated into the nomograms. All the nine variables were demonstrated to be independent prognostic factors for OS and BCSS. According to the point scale in these nomograms, each patient with different clinicopathologic characteristics could get a total point that can be used to predict the survival (1- and 3-year OS and BCSS). In addition, through comparing the survival outcomes predicted by those separate nomograms, we can also determine each patient's survival prognosis when performing surgical treatment or not. In general, a higher score was considered to have worse prognosis.

Subsequently, these individualized nomograms were validated internally and externally by calculating the C-index. For OS and BCSS in surgery group, the C-index were 0.721 (95% CI: 0.707-0.735) and 0.722 (95% CI: 0.708-0.736) in the internal validation, and 0.760 (95% CI: 0.730-0.790) and 0.770 (95% CI: 0.740-0.800) in the external validation, respectively, indicating a good predictive accuracies. Moreover, the corresponding C-index in non-surgery group were 0.664 (95% CI: 0.652-0.676), 0.666 (95% CI: 0.654-0.678), 0.692 (95% CI: 0.674-0.710) and 0.696 (95% CI: 0.677-0.715). In addition, the calibration curves plotted for these nomograms indicated a good correlation between the nomogram-predicted survival probability and the observed survival probability both in the training and validation set (Supplementary Figure 1).

### Preoperative Nomogram and External Validation

**Figure 4:**
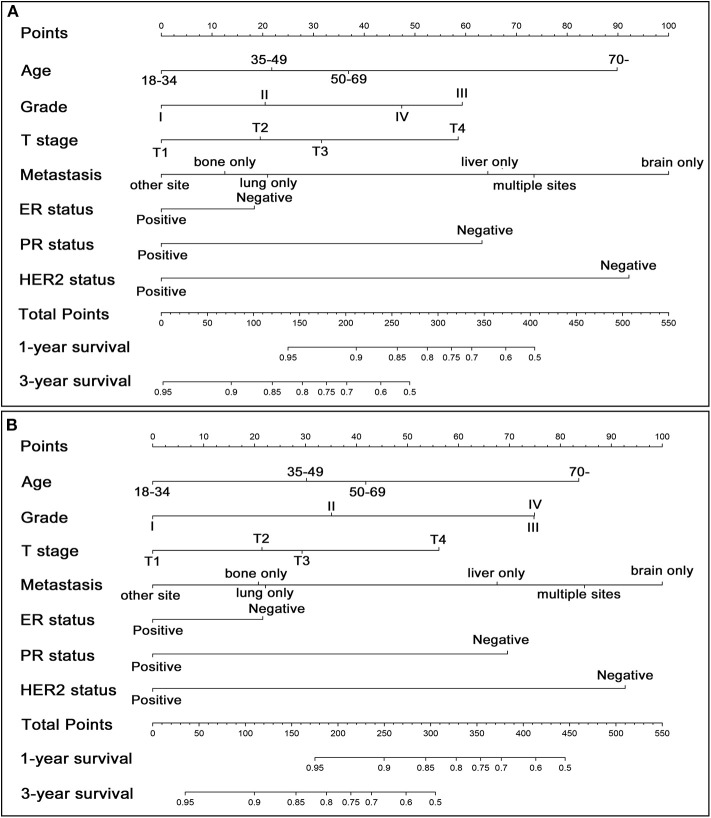
Preoperative nomogram for predicting 1- and 3-year OS **(A)** and BCSS **(B)** in patients with metastatic breast cancer who are candidate for surgical treatment.

Since it makes great sense to preoperatively assess whether patients could benefit from the surgical treatment, a preoperative nomogram was designed to predict survival benefit before making surgical decisions. Seven preoperatively measurable variables were included in the preoperative nomogram (Figure 4). As shown in Figures 4A,B, T stage and distant metastasis can be detected precisely by modern imaging techniques while information of grade, ER, PR and HER2 can be ascertained by aspiration biopsy. The C-index of the preoperative nomogram for OS using bootstrap and external validation were 0.713 (95% CI: 0.699-0.727) and 0.745 (95% CI: 0.714-0.776), respectively. A similar C-index for BCSS was also gained with 0.715 (95% CI: 0.701-0.729) in internal validation and 0.758 (95% CI: 0.727-0.789) in external validation, respectively. The calibration curves based on bootstrap resampling and validation set were shown in Supplementary Figure 2.

## Discussion

It is still somewhat controversial that whether patients with MBC can get survival benefits from performing locoregional surgical treatment. Amounts of retrospective studies have outlined clear benefits for MBC patients who had undergone surgical treatment (9, 15–18). For example, a retrospective study by Blanchard et al. indicated that the median survival of surgically treated MBC patients was significantly longer than patients without surgical resection in a multivariate analysis (*p* = 0.006) (19). Moreover, a recent meta-analysis included a large sample size of 67272 patients from 30 observational studies showed that primary tumor resection significantly improved not only OS (HR = 0.65, 95% CI: 0.61−0.70, *p* < 0.001) but also distant progression-free survival (HR = 0.42, 95% CI: 0.29−0.60, *p* < 0.001) (20). It was reported that surgical removal of primary tumor can reduce the tumor burden, remove the source of new metastases, and potentially reverse tumor-induced immunosuppression despite the presence of metastatic disease (21). However, a limited number of prospective randomized controlled clinical trials have yielded conflicting results. A randomized trial conducted in Turkey found that, compared with the initial systemic therapy group, patients in the initial surgery group had a significant reduction in the risk of death at 5 years, but not at 3 years (13). The stratified analysis also demonstrated that patients with HR positive, HER2 negative, younger age, or solitary bone-only metastases might be the potential subgroup who can benefit from surgical treatment. On the contrary, another randomized trial conducted in India found that tumor resection after a response to chemotherapy did not significantly improve overall survival (12). However, some selection biases existed in this study may confound the conclusion. Firstly, among all the patients with HER2 positive (35%) in this trial, only 15 percent received anti-HER2 therapy due to financial issues and none of it was included in local surgery group. Secondly, most patients enrolled in this study have developed clinical symptoms due to late diagnosis, making the median survival much lower than that in developed countries. Therefore, the study was unable to accurately assess the impact of surgery on the overall prognosis of patients receiving standard chemotherapy and targeted therapy. Importantly, the results of well-designed clinical trial, ECOG E2108 (NCT01242800) conducted in United States and Canada, were eagerly awaited to clarify the actual role of surgery in MBC patients.

Nowadays, metastatic breast cancer has been considered as a heterogeneous diseases. Survival rate of metastatic breast cancer have improved dramatically over the past few decades (18, 22). It was reported that the 5-year disease specific survival (DSS) of de novo MBC has improved from 28% (1990–1998) to 55% (2005–2010) (23). This could be attributed to early diagnosis with advanced imaging modalities and multiple modern systemic therapy with remarkable response rate, including endocrine therapy, anti-HER2 therapy, CDK4/6 inhibitor and mTOR inhibitor (24–26). Due to the prolonged survival of patients with metastatic breast cancer, we considered that selected subgroup of MBC could benefit from locoregional surgical treatment.

A large cohort of MBC patients diagnosed from 2010 to 2015 in SEER program were then analyzed in this study. Patients between 2010 and 2013 were selected as training set while patients diagnosed after 2013 as validation set. There were many different characteristics between the training set and validation set, which might enhance the credibility of our findings. It was notable that patients in validation set had received less radiation therapy as well as surgical intervention than patients in training set, which might be due to the formation of ideas that stage IV disease is not curative. In this situation, treatments with minimal harm are preferred to prolong survival and enhance quality of life (27). When analyzing the cohort of MBC patients diagnosed from 2010 to 2013, we found the administration of surgical treatment was significantly associated with better OS and BCSS, which was in consistent with the SEER based published studies analyzing the MBC patients between 1988 and 2011 (28). In the subgroup analysis, a multivariate cox analysis indicated that receiving surgery improved the OS and BCSS in almost all subgroups including patients with brain metastasis. This was different from a recent study implying that breast surgery provided no survival advantage for MBC patients with brain metastasis (29).

Since a systemic adjuvant therapy for MBC patients are still preferentially recommended by various guidelines (4, 30), we separately established univariate and multivariate Cox regression models both in surgery and non-surgery groups to identify survival-related risk factors, respectively. Our findings suggested that independent prognostic factors for worse OS and BCSS in both surgery and non-surgery cohort include older age, larger tumor size, positive HR and HER2 status, administration of radiation and chemotherapy, and the site of distant metastasis. Intriguingly, positive HER2 status, a well-known poor prognostic feature (31, 32), was proved to a protective factor in our study, largely because of widely usage of anti-HER2 therapy. Considering the uncertainty of survival benefit gotten from surgical treatment in IV stage breast cancer patients, nomograms predicting the long-term OS and BCSS with or without surgery would be useful to inform clinical decision making (33). Hence, several individualized nomograms were constructed in this study based on the result of multivariate Cox analysis. Our nomograms showed an acceptable predictive capabilities with C-index range of 0.65–0.80 both in training set and external validation set, which was comparable to some widely accepted nomograms (34–36).

We considered that a preoperative nomogram would be of great use when a untreated *de novo* metastatic breast cancer was diagnosed in patient with good performance or single/oligometastasis. Hence, a preoperative version of nomogram was designed by including seven preoperatively measurable variables. By means of aspiration biopsy, it is easy for surgeons to access information about ER, PR, HER2 and histological grade. Although T staging is usually determined postoperatively, a modern advanced imaging modalities, including breast magnetic resonance imaging (MRI), mammogram, ultrasound and Positron Emission Tomography-Computed Tomography (PET-CT), are supposed to provide precise assessment for tumor invasion and distant metastasis. Similarly, the bootstrap C-index above 0.70 both in training and validation set suggested a sufficient rate of accuracy. In addition, we hold the opinion that patient's state of health, expression level of Ki-67, ER and ER, and the effect of neoadjuvant chemotherapy would affect the clinician's surgical decision-making. Considering that those information were not available in SEER database, a large database containing detailed information of those variables mentioned above should be established and analyzed to further enhance the preoperative nomogram's predictive capability.

Inevitably, there are some limitations in our study. Firstly, the detailed information, such as regarding residues of tumor resection (R0, R1, or R2), endocrine therapy, sequence of chemotherapy, are not accessible in SEER database. All these factors were thought to have impact on survival of MBC patients who had undergone surgical treatment. Secondly, our study is retrospective and selection bias is inherent in the data that the MBC patients who received surgery or not were selected subjectively by the initial surgeon in the first place. We hold the opinion that a retrospective study cannot fully prove the advantage of surgery to metastatic breast cancer. The only way to investigate the exact role of locoregional surgical treatment in IV stage breast cancer would be a well-designed prospective randomized trial. Hence, we look forward to the ECOG E2108 and other ongoing clinical trials that may provide some valuable conclusions in future.

## Conclusions

This study suggests potential survival benefits of surgery among patients with metastatic breast cancer by analyzing population-based data. In addition, we constructed several individualized pre- and postoperative nomograms that are capable of predicting long-term survival of metastatic breast cancer patients with or without surgery, which may assist clinicians to make the appropriate treatment choices as well as to assess their patients' prognosis.

## Data Availability Statement

Publicly available datasets were analyzed in this study. This data can be found here: https://seer.cancer.gov/.

## Author Contributions

YZ and QY contributed to the idea and design. YZ, GZ, KY, KL, and QY contributed to the data acquisition and analysis. YZ and QY contributed to the manuscript writing and revision. All authors have read and approved the final version of this manuscript.

### Conflict of Interest

The authors declare that the research was conducted in the absence of any commercial or financial relationships that could be construed as a potential conflict of interest.
