# Corrigendum: Individualized Prediction of Survival Benefit From Locoregional Surgical Treatment for Patients With Metastatic Breast Cancer

**DOI:** 10.3389/fonc.2020.00482

**Published:** 2020-04-09

**Authors:** Yajuan Zheng, Guansheng Zhong, Kun Yu, Kefeng Lei, Qiong Yang

**Affiliations:** ^1^Department of Breast and Thyroid Surgery, Zhejiang Provincial People's Hospital, People's Hospital of Hangzhou Medical College, Hangzhou, China; ^2^Department of Breast Surgery, The First Affiliated Hospital, College of Medicine, Zhejiang University, Hangzhou, China; ^3^Department of General Surgery, The 7th Affiliated Hospital of Sun Yat-sen University, Shenzhen, China

**Keywords:** metastatic breast cancer, nomogram, SEER program, prognosis, clinic utility

In the published article, there was an error regarding the affiliation for “Kefeng Lei.” As well as having 3, Kefeng Lei should also have affiliation 1. In addition, there was a mistake in the order of corresponding authors. The first corresponding author should be Qiong Yang.

The authors apologize for these errors and state that this does not change the scientific conclusions of the article in any way. The original article has been updated.

