# Supplementary material for: Individualized Prediction of Survival Benefit From Locoregional Surgical Treatment for Patients With Metastatic Breast Cancer
Source: Front Oncol. 2020 Feb 18;10:148. doi: 10.3389/fonc.2020.00148 (PMC7040087; doi:10.3389/fonc.2020.00148)
Supplement: Supplementary file 1 [file Table_1.DOCX]

Supplementary Material

# Supplementary Figures and Tables

## Supplementary Figures


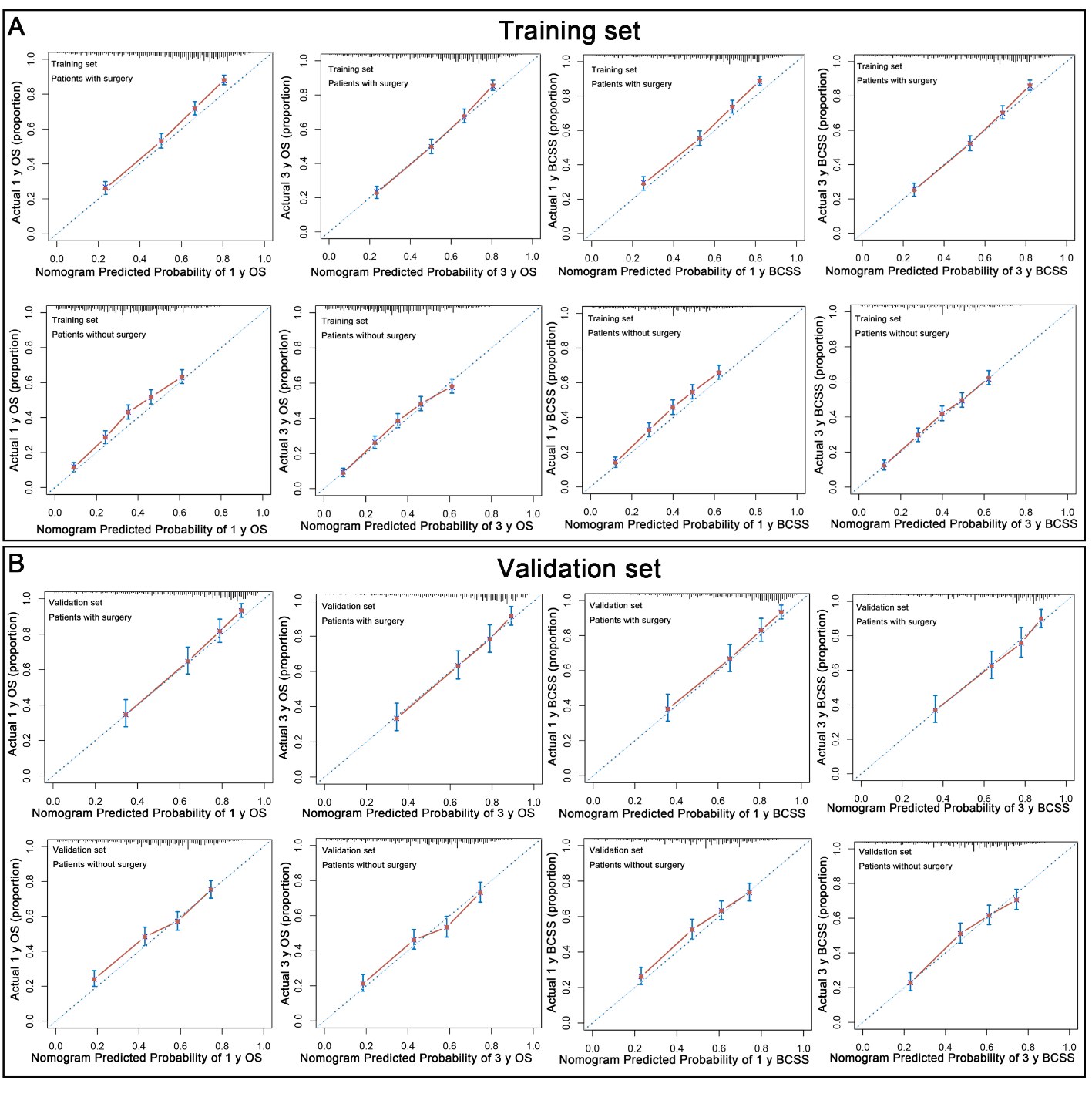


**Supplementary Figure 1.** Calibration curves for these nomograms in training set (A) and validation set (B). The 45-degree blue dotted line represents the ideal reference which means the nomogram-predicted survival probabilities (x-axis) exactly matches the actual survival proportions (y-axis). Red dots represent nomogram-predicted probabilities for each group, and blue error bars represent the 95% CIs of these estimates

**
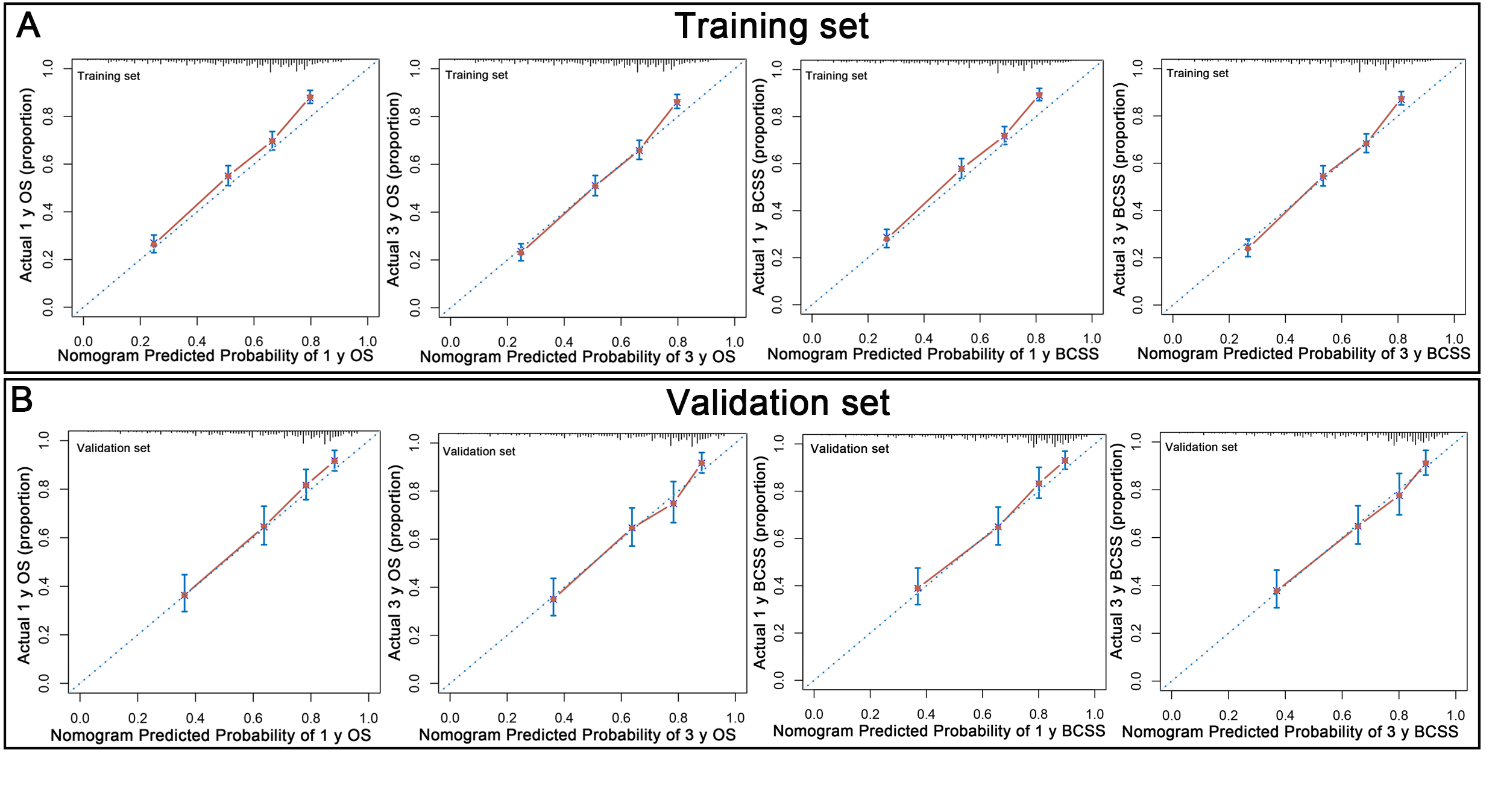
**

**Supplementary Figure 2.** Calibration curves for preoperative nomograms in training set (A) and validation set (B). The 45-degree blue dotted line represents the ideal reference which means the nomogram-predicted survival probabilities (x-axis) exactly matches the actual survival proportions (y-axis). Red dots represent nomogram-predicted probabilities for each group, and blue error bars represent the 95% CIs of these estimates.

## Supplementary Table

**Supplementary Table 1.** Difference of patient characteristics in training and validation set.

| **Variables** | Total population  [n (%), N= 8097] | Development set  [n (%), N= 5173] | Validation set  [n (%), N=2924] | P value |
| --- | --- | --- | --- | --- |
| **Age** |  |  |  | 0.040 |
| **< 35** | 339 (4.2) | 201 (3.9) | 138 (4.7) |  |
| **35-49** | 1616 (20.0) | 1070 (20.7) | 546 (18.7) |  |
| **50-69** | 4199 (51.9) | 2686 (51.9) | 1513 (51.7) |  |
| **≥ 70** | 1943 (24.0) | 1216 (23.5) | 727 (24.9) |  |
| **Gender** |  |  |  | 0.345 |
| **Female** | 7996 (98.8) | 5113 (98.8) | 2883 (98.6) |  |
| **Male** | 101 (1.2) | 60 (1.2) | 41 (1.4) |  |
| **Race** |  |  |  | 0.024 |
| **Black** | 1424 (17.6) | 885 (17.1) | 539 (18.4) |  |
| **White** | 6009 (74.2) | 3888 (75.2) | 2121 (72.5) |  |
| **Other** | 664 (8.2) | 400 (7.7) | 264 (9.0) |  |
| **T stage** |  |  |  | 0.209 |
| **T1** | 931 (11.5) | 612 (11.8) | 319 (10.9) |  |
| **T2** | 2779 (34.3) | 1785 (34.5) | 994 (34.0) |  |
| **T3** | 1498 (18.5) | 925 (17.9) | 573 (19.6) |  |
| **T4** | 2889 (35.7) | 1851 (35.8) | 1038 (35.5) |  |
| **N stage** |  |  |  | 0.012 |
| **N0** | 1684 (20.8) | 1090 (21.1) | 594 (20.3) |  |
| **N1** | 3857 (47.6) | 2398 (46.4) | 1459 (49.9) |  |
| **N2** | 1099 (13.6) | 736 (14.2) | 363 (12.4) |  |
| **N3** | 1457 (18.0) | 949 (18.3) | 508 (17.4) |  |
| **Grade** |  |  |  | 0.027 |
| **High, I** | 568 (7.0) | 356 (6.9) | 212 (7.3) |  |
| **Intermediate, II** | 3305 (40.8) | 2079 (40.2) | 1226 (41.9) |  |
| **Low, III** | 4172 (51.5) | 2696 (52.1) | 1476 (50.5) |  |
| **Anaplastic, IV** | 52 (0.6) | 42 (0.8) | 10 (0.3) |  |
| **Distant metastasis** |  |  |  | 0.138 |
| **Bone only** | 3058 (37.8) | 1944 (37.6) | 1114 (38.1) |  |
| **Liver only** | 624 (7.7) | 397 (7.7) | 227 (7.8) |  |
| **Lung only** | 851 (10.5) | 515 (10.0) | 336 (11.5) |  |
| **Brain only** | 90 (1.1) | 54 (1.0) | 36 (1.2) |  |
| **Other site** | 958 (11.8) | 637 (12.3) | 321 (11.0) |  |
| **Multiple sites** | 2516 (31.1) | 1626 (31.4) | 890 (30.4) |  |
| **ER status** |  |  |  | 0.836 |
| **Negative** | 2121 (26.2) | 1359 (26.3) | 762 (26.1) |  |
| **Positive** | 5976 (73.8) | 3814 (73.7) | 2162 (73.9) |  |
| **PR status** |  |  |  | 0.236 |
| **Negative** | 3254 (40.2) | 2104 (40.7) | 1150 (39.3) |  |
| **Positive** | 4843 (59.8) | 3069 (59.3) | 1774 (60.7) |  |
| **HER2 status** |  |  |  | 0.013 |
| **Negative** | 5816 (71.8) | 3764 (72.8) | 2052 (70.2) |  |
| **Positive** | 2281 (28.2) | 1409 (27.2) | 872 (29.8) |  |
| **Radiation** |  |  |  | <0.001 |
| **No** | 5153 (63.6) | 3200 (61.9) | 1953 (66.8) |  |
| **Yes** | 2944 (36.4) | 1973 (38.1) | 971 (33.2) |  |
| **Chemotherapy** |  |  |  | 0.094 |
| **No** | 3127 (38.6) | 2033 (39.3) | 1094 (37.4) |  |
| **Yes** | 4970 (61.4) | 3140 (60.7) | 1830 (62.6) |  |
| **Surgery type** |  |  |  | <0.001 |
| **No surgery** | 4965 (61.3) | 2970 (57.4) | 1995 (68.2) |  |
| **Lumpectomy or mastectomy** | 1597 (19.7) | 1121 (21.7) | 476 (16.3) |  |
